# Asymptomatic central venous occlusion secondary to central venous catheter-use complicating pacemaker implantation: a leadless solution

**DOI:** 10.1093/omcr/omaf292

**Published:** 2026-01-25

**Authors:** James Baudry, Christopher Cassidy, Kanarath Balachandran

**Affiliations:** Cardiology, East Lancashire Hospital NHS Trust (Royal Blackburn Hospital, Haslingden Road, Blackburn BB2 3HH, United Kingdom); Cardiology, Blackpool NHS Teaching Hospitals (Blackpool Victoria Hospital, Whinney Heys Road, Blackpool FY3 8NR, United Kingdom); Cardiology, East Lancashire Hospital NHS Trust (Royal Blackburn Hospital, Haslingden Road, Blackburn BB2 3HH, United Kingdom)

**Keywords:** complete-heart-block, venous occlusion, central venous catheter, cardiac resynchronisation therapy, Micra-AV, leadless pacing

## Abstract

Abnormal central venous anatomy can obstruct cardiac-device implantation. We report a 54-year-old patient found to be in complete heart-block after identification of profound bradycardia, requiring cardiac-pacing. Echocardiography revealed systolic dysfunction, prompting a plan for cardiac resynchronization therapy. Conventional lead placement was impossible due to failed guide-wire advancement bilaterally. Peri-procedural venography demonstrated attenuation of both brachiocephalic veins and contrast-enhanced computed tomography confirmed complete central venous occlusion (CVO). Further history identified previous chemotherapy delivered via central venous catheters, as the likely aetiology for the CVO *and* systolic dysfunction. Alternative pacing options were considered. Ultimately the leadless *Micra-AV*-pacemaker was successfully implanted via the femoral vein with good clinical recovery. This unique case highlights CVO as an obstacle to cardiac-device implantation, with specific patients at increased risk. Pre-procedural imaging in those at-risk may reduce procedure failure and facilitate appropriate device strategy choice. Leadless pacing provides a safe, effective alternative in cases of CVO.

## Introduction

Cardiac implantable devices (CIDs) are usually inserted via the central thoracic veins [1]. In some patients, unrecognized venous abnormalities preclude access. These abnormalities are often asymptomatic and therefore not detected pre-procedurally, frequently leading to procedure abandonment or delay [2, 3].

While congenital anomalies are well documented, acquired central venous occlusions (CVOs) are less well reported [2]. Importantly, patients requiring CIDs are at higher inherent risk of CVOs, however their impact is poorly characterised [3–5].

We present a unique case of asymptomatic, chemotherapy-associated CVO precluding standard transvenous-lead access. We emphasize the diagnostic and procedural challenges of CVOs, and introduce the role of leadless pacing as a practical pacing alternative [6].

## Case presentation

**Figure 1:**
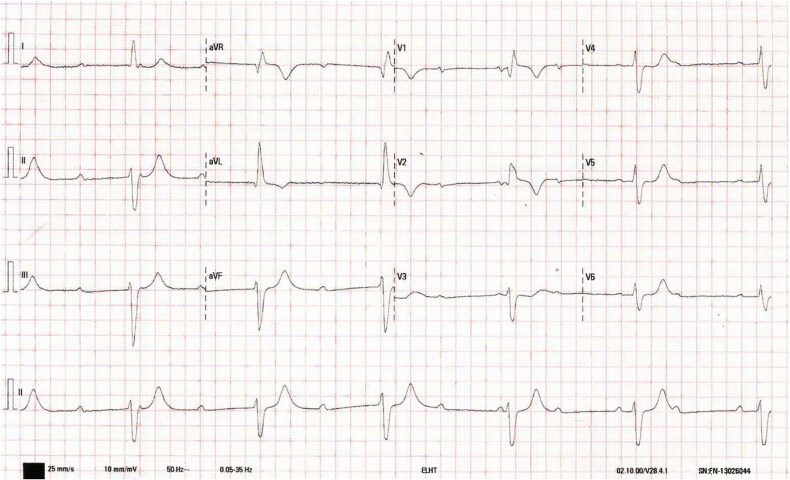
*Twelve-lead electrocardiogram on admission.* The tracing demonstrates complete atrioventricular dissociation, with atrial complexes occurring at approximately 70 beats per minute and broad, regular ventricular complexes at 35 beats per minute (QRS duration 162 milliseconds). The tracing is consistent with complete (third-degree) atrioventricular block. This conduction disease requires cardiac pacing.

A 54-year-old white British man presented to the emergency department with a hot, swollen knee. Triage revealed profound bradycardia of 35 bpm. History highlighted one episode of dizziness two weeks earlier and non-specific fatigue for one month. Apart from bradycardia, examination was unremarkable. Electrocardiogram showed broad (162 ms) QRS complexes in third-degree atrio-ventricular (AV) Block (Fig. 1). He was admitted to the cardiology ward for urgent cardiac pacemaker insertion and monitored on telemetry.

Pre-procedural transthoracic echocardiography revealed left ventricular (LV) systolic dysfunction (Ejection Fraction 30–35%). Given the combination of LV dysfunction and the need for pacing, Cardiac Resynchronization Therapy (CRT) was recommended over standard pacing. The underlying cause of conduction and structural abnormalities in this relatively young patient was not yet established, but device implantation was prioritized.

**Figures 2:**
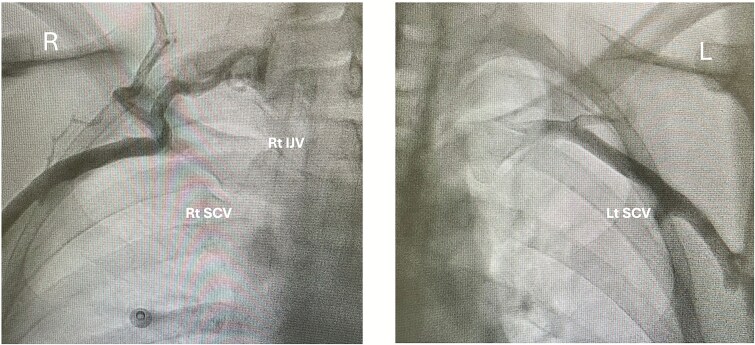
A–B. *Fluoroscopic venography of the right (A) and left (B) upper thoracic venous systems.* (A) Contrast opacifies the right cephalic vein with progression into the axillary vein and limited opacification of the right subclavian vein. There is no contrast passage into the expected location of the right brachiocephalic vein or superior vena cava (SVC), findings consistent with complete right-sided central venous occlusion. Multiple prominent collateral vessels—Most likely representing the external jugular and cervical venous collaterals—Are seen superior to the subclavian vein, reflecting diversion of flow through compensatory channels. (B) Contrast opacifies the left cephalic, axillary, and proximal subclavian veins; however, there is no contrast progression into the left brachiocephalic vein or SVC, in keeping with complete occlusion of the left central venous outflow. Both images are in keeping with bilateral central venous occlusion (CVO).

CRT-P implantation was first attempted via the left subclavian vein. On guidewire insertion however, resistance was encountered, preventing advancement. Bedside venography revealed attenuation of the left brachiocephalic vein (Fig. 2A). Access was then attempted on the right but this was unsuccessful. Venography demonstrated similar attenuation of the right brachiocephalic vein (Fig. 2B). The procedure was abandoned pending further imaging.

**Figures 3:**
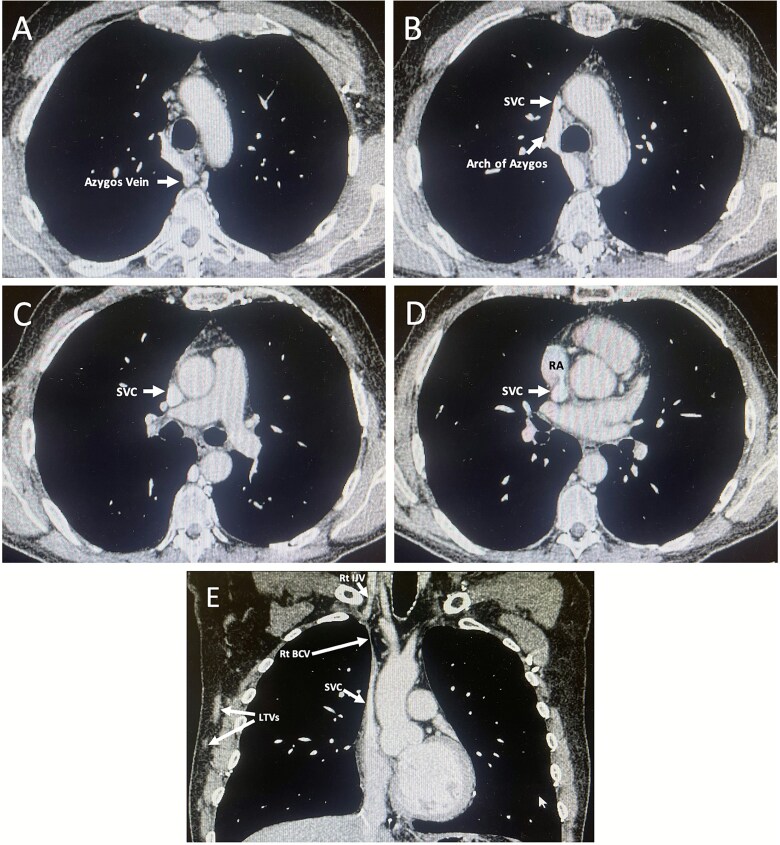
*A-E: Contrast-enhanced CT of the thorax (venous phase). Transverse sections are shown in cranio–caudal order (A–D), with a coronal reconstruction (E).* (A) Axial image at the level of the aortic arch demonstrates a markedly dilated azygos vein draining into a prominent arch of azygos. The superior vena cava (SVC) is not visualised at this level, consistent with complete occlusion. The brachiocephalic veins—Which normally converge to form the SVC at this level—Are extremely difficult to appreciate, in keeping with non-opacification due to underlying occlusion. (B) Axial image immediately caudal to (A) again shows a prominent arch of azygos draining into a severely stenotic upper SVC. (C) Axial image at the level of the pulmonary trunk bifurcation demonstrating persistent SVC narrowing. (D) Axial image at the SVC–right atrial (RA) junction, where the SVC is patent. (E) Coronal reconstruction demonstrating a patent right internal jugular vein (IJV), complete occlusion of the right brachiocephalic vein (BCV), and non-opacification of the left IJV and left BCV consistent with chronic occlusion. Severe upper SVC stenosis is present. Prominent right-sided lateral thoracic veins (LTVs) are visible, reflecting collateral venous drainage of the right upper limb.

Contrast-enhanced computed tomography (CT) confirmed complete occlusion of both brachiocephalic veins, the left internal jugular vein, and the upper SVC (Fig. 3A-E). All upper-limb venous drainage occurred through collateral pathways primarily via the azygous vein (Fig. 3E). The occlusions appeared chronic and no evidence of malignancy was identified. A differential diagnosis of Paget-Shrötters was considered. Further history taking revealed that the patient had received chemo-radiotherapy for Grade 3B Hodgkin lymphoma 33 years prior. Specialist radiological review found minimal radiation-related change. The occlusions were therefore attributed to long-term CVC-use for chemotherapy. Anthracycline exposure was considered the likely cause of LV dysfunction and conduction abnormalities.

**Figure 4:**
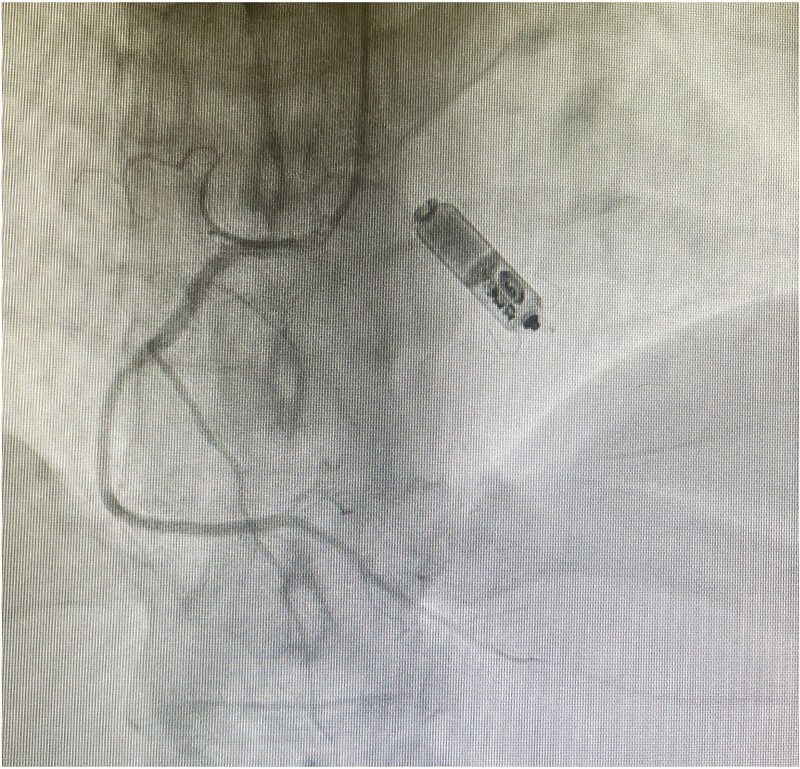
*Fluoroscopic PA-cranial 30° projection from invasive coronary angiography.* Leadless Micra-AV pacemaker in a stable position within the right ventricular cavity. Concurrent right coronary artery (RCA) angiography shows a normal, unobstructed vessel.

Impossible thoracic central-venous access prompted referral for alternative pacing-strategies. Femoral CRT was rejected owing to infection and lead-displacement risk, and epicardial pacing was deemed unsuitable due to prior chest irradiation. The multidisciplinary team elected to implant a leadless pacemaker (Micra-AV) via femoral route. This would provide AV-synchrony, albeit without ventricular resynchronisation. Implantation was successful on first deployment with satisfactory pacing parameters (Fig. 4). The patient recovered well, with good pacing function follow-up and optimisation of heart-failure pharmacotherapy.

## Discussion

This case illustrates complete heart block with LV dysfunction in a relatively young patient where bilateral CVOs, likely secondary to prior chemotherapy via CVC, precluded conventional CRT implantation. Anthracycline chemotherapy-agents were likely responsible for both conduction abnormalities and systolic dysfunction, given their association with both [7].

### Differential diagnoses for abnormal central veins

Central venous abnormalities may be congenital or acquired, the latter being more common [2]. Congenital variants are rare and well described in paediatric cardiology, they are therefore not discussed further here [2]. *Acquired* abnormalities usually represent CVOs and arise from either external compression or intraluminal venous conditions. Clinical presentation depends on the speed of onset: chronic occlusions are often asymptomatic and therefore unrecognized until central-access is sought [2–4].

External compression most often results from malignant mediastinal masses [2, 8], while musculoskeletal causes such as Paget-Schrötter syndrome are rare [9]. In this case, CT findings excluded these. Iatrogenic CVOs are more common, commonly occurring after CVC or CID lead insertion, both of which provoke endothelial injury, thrombosis and eventual stenosis-occlusion [2, 4]. Reported rates of CVO or severe stenosis after CID implantation range from 20–30% [4, 10]. Among patients using CVCs, haemodialysis patients are most affected with occlusion-rates approaching 50%; chemotherapy delivery via CVC also increases CVO risk, hence the explanation for occlusion in this patient [2, 3].

### Impact of CVO on CIDs

The impact of CVOs on CID implantation is underexplored, with most literature focusing on occlusions developing *after* device placement [4]. Nevertheless, pre-existing CVOs in those undergoing CID implantation are not uncommon: one retrospective study reported pre-procedural occlusions of 14% [5]. The relatively high prevalence of CVOs in this population reflects overlapping risk factors: a high proportion of prior transvenous CID leads *and* CVC exposure. Both prior transvenous leads *and* the use of CVCs for chemotherapy or haemodialysis increase CVO risk, while these same patients are also predisposed to cardiovascular disease, itself requiring device therapy. For example, 6–7% of haemodialysis patients require a CID, and many patients require revision procedures for previously implanted CIDs [4, 10].

Current guidance recommends pre-procedural venous imaging only when obstruction is clinically suspected [1]. Consequently, and, as seen in this case, asymptomatic CVOs are missed. This can result in procedural difficulties, patient distress and delayed therapy. Impossible conventional access prompts consideration of alternative pacing strategies.

### Leadless pacing

Alternative pacing strategies include epicardial pacing, femoral systems and leadless devices [1]. Epicardial and femoral systems are viable but carry risks of surgical morbidity and lead displacement, respectively, as well as infection [6]. Leadless pacemakers, implanted directly into the endocardium via femoral approach, provide a minimally invasive alternative, and are now advocated when thoracic-access is limited [1]. Current data suggests superior pacing parameters and safety compared with epicardial systems [11]. In one study, haemodialysis patients were found to have better long-term outcomes with leadless-devices than thoracic systems [10].

The Micra-AV used in this case employs surrogate atrial sensing to maintain reasonable AV synchrony_._ The newer Aveir dual-chamber device achieves true synchrony through the addition of a right-atrial device. This was unavailable at the time. No current options provide leadless *biventricular* pacing suitable for CRT, though emerging conduction-system pacing may expand indications [11].

### Novelty and importance

Few reports describe leadless pacing to overcome abnormal venous anatomy. A similar case is reported in which SVC-obstruction secondary to malignant lymphoma precluded conventional transvenous-leads, and was overcome by femoral Micra-AV insertion [8]. To our knowledge however, our case represents the first use of leadless-pacing in *asymptomatic CVO* secondary to CVC-delivered chemotherapy; rather than symptomatic malignancy-related venous-occlusion. This case highlights how CVOs, especially those without clinical-signs, can impact CID insertion, and that pre-procedural imaging may be justified in high-risk patients. Leadless pacing in patients with CVO offers a safe, effective alternative with increasing applications.

## Conclusion

Central venous abnormalities are an under-recognized obstacle to cardiac device implantation. CVOs represent the most clinically important of these abnormalities and often go undetected pre-procedurally, negatively impacting procedure success. Early identification of at-risk patients with prior: CVC-use (for chemotherapy or haemodialysis), or CIDs, can help anticipate risk. Pre-procedural imaging and early consideration of alternative pacing approaches in these patients can minimise disruption. Leadless pacing in particular offers a safe, effective alternative with good clinical outcomes.
